# Ectopic papillary thyroid carcinoma in the mediastinum without any tumoral involvement in the thyroid gland. A Case report

**DOI:** 10.7508/aojnmb.2013.01.009

**Published:** 2013

**Authors:** Susan Shafiee, Ali Sadrizade, Amirhosein Jafarian, Seyed Rasoul Zakavi, Narjess Ayati

**Affiliations:** 1 Nuclear Medicine Research Center, Mashhad University of Medical Sciences, Mashhad, Iran; 2 Thoracic Surgery department, Mashhad University of Medical Sciences, Mashhad, Iran; 3 Cancer biology research Center, Mashhad University of Medical Sciences, Mashhad, Iran

**Keywords:** Papillary thyroid carcinoma, Ectopic PTC, Ectopic thyroid, mediastinal mass

## Abstract

Ectopic thyroid tissue results from abnormal embryologic development and migration of the thyroid gland. True malignant transformation in ectopic thyroid tissue is extremely rare and is always diagnosed after surgical excision of the lesion by pathology examinations. There are well-documented cases of ectopic thyroid cancer while primary tumoral lesion occurs in the orthotopic thyroid, but only rare cases of ectopic PTC without any evidence of occult thyroid cancer in the orthotopic thyroid or cervical lymph nodes have been reported. We report on a 39 year old woman who was operated for a mediastinal mass. The initial diagnosis was a malignant thymic lesion, which was later confirmed to be a papillary thyroid carcinoma. Consequently, total thyroidectomy was performed and pathology report showed normal thyroid tissue with no evidence of any neoplastic involvement. Until now, only one similar case has been reported.

## Introduction

Ectopic thyroid tissue is a common abnormality and results from abnormal embryologic development and migration of the thyroid gland (1). It is mainly found at the base of the tongue while the thoracic cavity is the most common non-cervical location for ectopic thyroid tissue (2-3).

True malignant transformation of ectopic thyroid tissue is extremely rare and is always diagnosed after surgical excision of the lesion by pathologic examination (4-5).

It is often difficult to determine whether a cancer in the ectopic thyroid tissue represents metastatic thyroid carcinoma with an undetected primary tumor or an ectopic thyroid carcinoma arising in the ectopic thyroid (2,6). There are rare cases of ectopic PTC without any evidence of occult thyroid cancer in the orthotopic thyroid or cervical lymph nodes suggesting a de novo process (2,6-8).

Although there are a few reports on ectopic PTC in sub mental, thyroglossal duct cysts and bronchial cleft cysts with normal pathologic examinations of orthotopic thyroid (2-3, 7-8), only one case of mediastinal PTC without any neoplastic involvement of the orthotopic thyroid gland has been reported (5).

## Case Report

**Figure 1:**
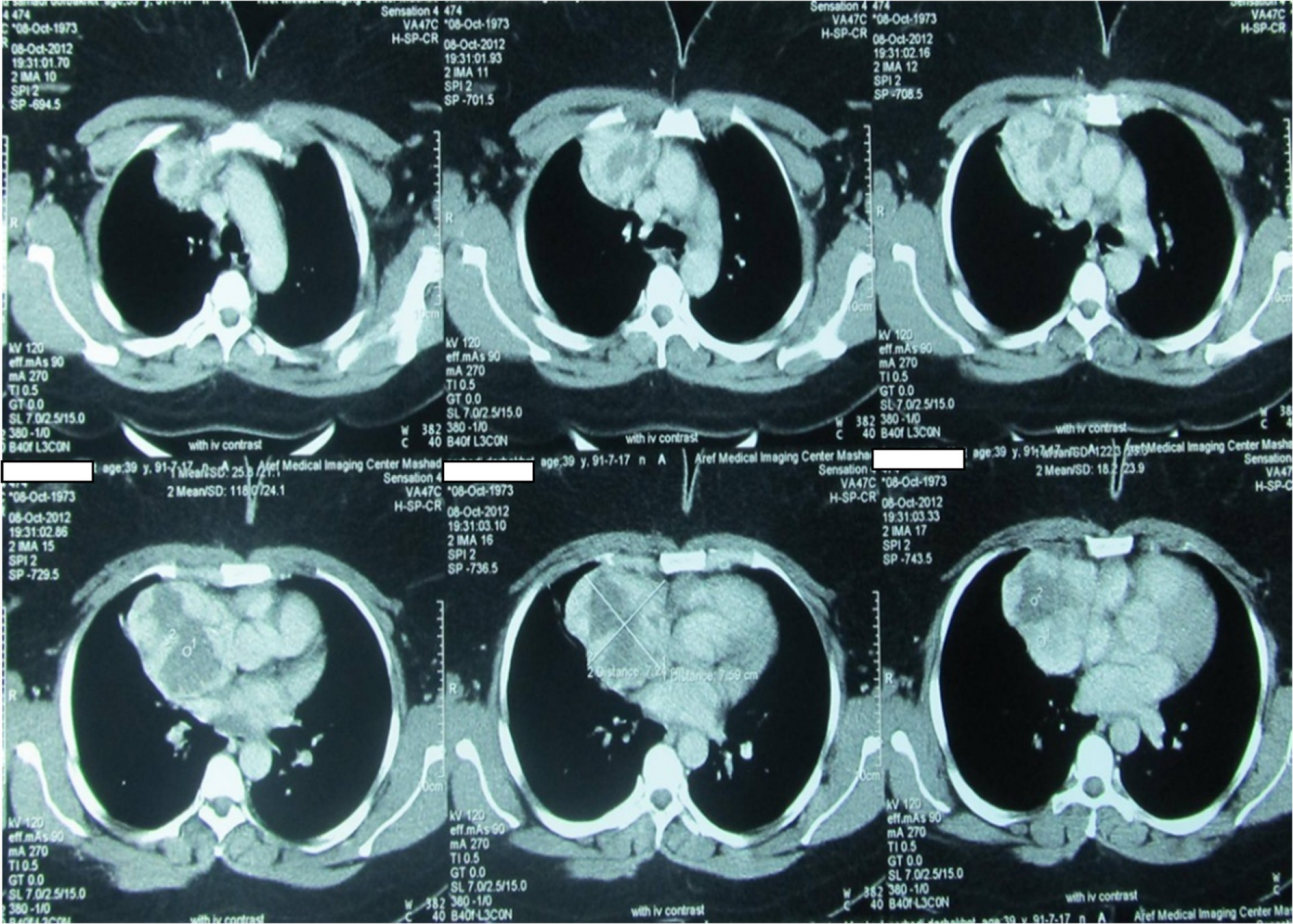
Multislices thoracic CT in axial slices shows mediastinal mass extending from suprasternal notch to above liver.

A 39 years old woman was admitted to the hospital because of left hemi thorax pain for two months. Physical examination was normal and a mediastinal mass was noted on a chest X Ray (CXR). Thoracic High Resolution CT (HRCT) revealed an 8cm lobulated mass with central hypo density extending from right side of the aortic pouch (adjacent to SVC) to the middle lobe of the right lung suggesting a malignant mass of thymic origin (figure 1). Bronchoscopic evaluation showed an external compression due to the mediastinal tumor at the orifice of the right upper lobe with no endobronchial lesion, and cytology of bronchial fluid was negative for malignancy.

**Figure 2:**
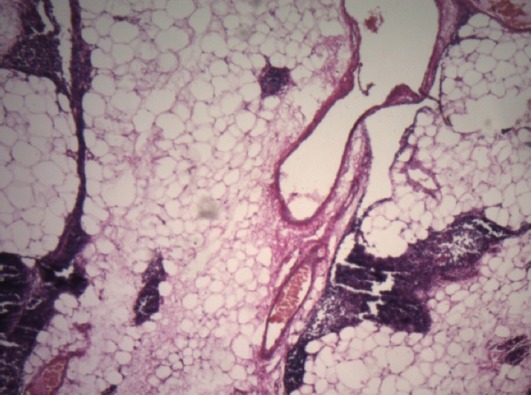
Pathologic diagnosis of PTC in mediastinal mass with nuclear staining IHC and TTF1 (×40).

**Figure 3:**
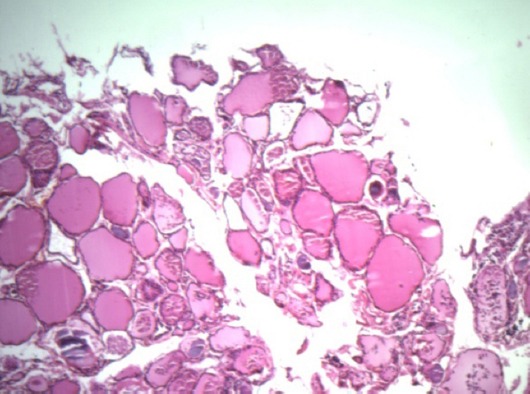
Normal thyroid gland cells with colloid achieved post thyroidectomy

Intraoperative evaluation of mediastinal mass showed no glandular continuity between the thyroid gland and the mass and blood supply of the mass was entirely derived from the vascular branches of thoracic vessels. Histopathology examination of the mass showed a papillary thyroid carcinoma. Immunohistochemistry studies confirmed the diagnosis and TTF1 was positive (figure 2). Thymus was atrophic and without any neoplastic involvement. On physical examination, thyroid was normal and no lymph node was palpable in the neck. Ultrasonographic examination showed a normal sized thyroid (Right lobe: 38×13×11 mm with 3 cc volume, Left lobe: 38×13×12 mm with 3.3 cc volume, and normal isthmus): with a 2.5 mm colloid cyst in the right lobe. Total thyroidectomy was performed and histopathologic evaluation showed a normal thyroid tissue without tumoral involvement (figure 3).

## Discussion

True malignant transformation of ectopic thyroid tissue is extremely rare. Most of the cases are characterized by papillary carcinomas. It is often difficult to determine whether a cancer in the ectopic thyroid tissue represents metastatic thyroid carcinoma with an undetected primary tumor or ectopic thyroid carcinoma arising in the ectopic thyroid (2,6). Both clinical situations have been described in the literature (5).

There are well-documented cases of ectopic thyroid cancer with a primary tumoral lesion present in the orthotopic thyroid, but only rarely have cases of ectopic PTC without any evidence of occult thyroid cancer in the orthotopic thyroid or cervical lymph nodes (suggesting a de novo process) been reported (2,6-8).

A few cases have been reported with ectopic PTC in sub mental, thyroglossal duct cysts and bronchial cleft cysts without any evidence of occult thyroid cancer in the orthotopic thyroid (2-3,5,7-8). In Medline there is only one reported case of mediastinal PTC without any neoplastic involvement in the orthotopic thyroid tissue (5).

There are few hypotheses discussing the etiology of such ectopic tissues. First and the most important explanation is metastatic lesion of papillary thyroid carcinoma. Another possibility is that a thyroid nodule may become detached from the gland. Also rarely there are ectopic thyroid masses which do not have features consistent with the above mentioned explanations (1).

This clinical report presents a case with mediastinal ectopic thyroid without orthotopic thyroid gland pathology. In this situation, the primary treatment after mediastinal mass resection is total thyroidectomy (1). Complete thyroid resection, not only excludes the thyroid gland as the primary source of malignancy but also facilitates future patient management by serum thyroglobolin level measurement and whole body iodine scintigraphy.

Mediastinal ectopic thyroid carcinoma is extremely rare. On careful review, the vast majority of mediastinal goiters will not fulfill the criteria and are found to be secondary goiters, or merely a retrosternal extension of a cervical goiter. Our case had a true ectopic mediastinal thyroid tissue as it derived its blood supply from intrathoracic vessels rather than cervical arteries and the thyroid gland had a normal morphology and histology.

This case report shows that a normal thyroid gland cannot exclude PTC in ectopic thyroid tissue and precise follow up of these patients is highly recommended.
